# Primary Care Networks and Starfield’s 4Cs: A Case for Enhanced Chronic Disease Management

**DOI:** 10.3390/ijerph18062926

**Published:** 2021-03-12

**Authors:** Chuan De Foo, Shilpa Surendran, Geronimo Jimenez, John Pastor Ansah, David Bruce Matchar, Gerald Choon Huat Koh

**Affiliations:** 1 Department of Health Systems and Behavioural Sciences, Saw Swee Hock School of Public Health, National University Singapore, Singapore 117549, Singapore; ephsuren@nus.edu.sg (S.S.); ephkohch@nus.edu.sg (G.C.H.K.); 2 Centre for Population Health Sciences (CePHaS), Lee Kong Chian School of Medicine, Nanyang Technological University, Singapore 308232, Singapore; geronimo.jimenez@gmail.com; 3 Department of Public Health and Primary Care, Leiden University Medical Center, 2333 ZA Leiden, The Netherlands; 4 Health Services and Systems Research, Duke-National University of Singapore Graduate Medical School, Singapore 169857, Singapore; john.ansah@duke-nus.edu.sg (J.P.A.); david.matchar@duke-nus.edu.sg (D.B.M.); 5 Department of Medicine, Division of General Internal Medicine, Duke University School of Medicine, Durham, NC 27710, USA

**Keywords:** qualitative, Starfield, 4Cs, chronic disease management, primary health care

## Abstract

The primary care network (PCN) was implemented as a healthcare delivery model which organises private general practitioners (GPs) into groups and furnished with a certain level of resources for chronic disease management. A secondary qualitative analysis was conducted with data from an earlier study exploring facilitators and barriers GPs enrolled in PCN’s face in chronic disease management. The objective of this study is to map features of PCN to Starfield’s “4Cs” framework. The “4Cs” of primary care—comprehensiveness, first contact access, coordination and continuity—offer high-quality design options for chronic disease management. Interview transcripts of GPs (n = 30) from the original study were purposefully selected. Provision of ancillary services, manpower, a chronic disease registry and extended operating hours of GP practices demonstrated PCN’s empowering features that fulfil the “4Cs”. On the contrary, operational challenges such as the lack of an integrated electronic medical record and disproportionate GP payment structures limit PCNs from maximising the “4Cs”. However, the enabling features mentioned above outweighs the shortfalls in all important aspects of delivering optimal chronic disease care. Therefore, even though PCN is in its early stage of development, it has shown to be well poised to steer GPs towards enhanced chronic disease management.

## 1. Introduction

As the world ages at a rapid pace, the number of patients with chronic conditions is set to increase in tandem. Chronic diseases are commonly characterised by their requirement for long term follow-up with healthcare providers, association with functional impairment or disability and need for holistic management of the patient [1]. Inevitably, the uptick in chronic disease load had led to an overwhelming burden on healthcare infrastructure and national health expenditures [2,3,4]. This affliction is accrued from the systematic stress precipitated by higher bed occupancies, hospital readmission numbers and emergency medicine interventions [5,6,7]. This perpetuating strain has created the catalyst to provide chronic disease management services for stable patients at the community level in order to free up health care resources at the tertiary care interface [8].

Therefore, the defining features of primary care which encompass comprehensiveness, continuity and coordination make this setting well equipped for community management of patients with chronic diseases [9]. Importantly, shifting stable chronic cases to the primary care space for long term management is timely and cogent [10]. Multiple studies had elucidated that health service expenditure reduction and overall more equitable health outcomes are derived when patients with chronic conditions are firmly anchored with their primary care providers [11,12,13]. Essentially, prescient health policy manoeuvres that focus on enhancing primary care capacities for chronic disease management will generate multi-fold benefits for the healthcare system and should be explored in detail.

### 1.1. Singapore’s Primary Care Landscape

For many countries, primary healthcare is heralded as the bedrock of their healthcare systems, and Singapore is no exception. Although, in 2014, Singapore was ranked first for its efficiency in healthcare delivery, the strength of its primary care did not fare well when healthcare experts applied Barbara Starfield’s seminal primary care framework [14,15]. Therefore, being ranked as a country with one of the highest life expectancies in the world, with a greying population forecasted to swell to one in four by 2030 and one in two by 2050, Singapore needs to upend and strengthen its primary healthcare system so as to contain tertiary healthcare utilisation, improve continuity of care and overall health outcomes [16,17,18].

At present, Singapore’s primary care sector is divided between private general practitioners (GPs) and polyclinics which are government-subvented primary care entities. Polyclinics are multi-doctor (usually more than 10) clinics that provide a comprehensive range of services for the family, functioning as a one-stop care centre for acute and chronic conditions. In contrast, private GPs are solo practices with limited services. Currently, 1700 private GP clinics and 20 polyclinics operate island-wide [19].

In terms of primary care utilisation, private GPs account for 80% of all primary care attendances, out of which only 20% are for chronic disease management [20]. On the other hand, polyclinics fulfil 52% of chronic disease attendances, while government tertiary hospitals manage the remaining [20]. With the imbalance in chronic disease attendances afflicting the polyclinics and tertiary sectors coupled with an increasing chronic disease burden, the primary care networks (PCNs) were commissioned by the Ministry of Health (MOH) as an enhanced mainstream primary care model in 2018 to mobilise more private primary care sector resources [21]. This was envisaged through the voluntary enrolment of GP practices into a PCN of their choice. As of November 2020, a total of 561 private GP practices have been enrolled [22]. In the PCN architecture, private GP practices are given the autonomy to allocate financial and manpower resources provided by MOH and dictate the internal workings of the network with two GP leaders at the helm. Such a ground-up approach to the PCN model enabled operational variegation to suit the specific characteristics of the different networks of GPs and their patients. Currently, there are ten PCNs [22]; each a cluster of hitherto independent and separate GP practices working more closely together to offer extended care services for patients with complex chronic conditions.

### 1.2. Starfield’s “4Cs” of Primary Care

There is a general agreement in primary care that the four core pillars enshrined in Starfield’s “4Cs” primary care framework, or more commonly referred to as the “4Cs”, are associated with patient-centred services, cost containment, reduction in unequal access to medical care and an overall improvement in population health [12,23]. Starfield explicated these four attributes as:

C1: “Comprehensive care”, which expands on the availability of a wide range of services in primary care to cater to a spectrum of health conditions [24]. Offering a comprehensive scope of services permits a practice to provide health promotion, prevention, diagnosis and treatment services for a range of health conditions throughout a patient’s life course [25]. This can be enabled by building teams of professionals including GPs, registered nurses and allied health professionals that are based in the primary care space [26]. Primary care teams can reduce the need for specialist referrals and services particularly when specialty services are made accessible at the primary care level. If the services are unavailable, the team can refer patients to community-based options to augment the existing primary care suite of services to provide sufficient breadth and depth of service coverage for holistic patient-centred care in the community [27].

C2: “First Contact of care” which refers to care first sought from the primary care provider (i.e., services must be accessible and used by the population each time new health or medical need arises) [24]. Broadly, first contact access is widely associated with primary care providers being the main point of entry and subsequently, the preferred point of contact with the health system. First contact opens the doors to the rest of the health system and coordination with other health entities permits that, especially when a gatekeeper role is mandated [28]. If prompt management of complications is required, GPs will consequently refer patients to higher levels of care. This has been shown to reduce overuse of specialist hospital resources and in turn, containment of ambulatory care expenditure [29].

C3: “Coordination of care”, which discusses the linking of health care visits and services so that patients receive appropriate care for all their health problems [24]. Chronic diseases often requisites a wide range of services, some of which fall outside the ambit of primary care. This makes the transition of patients across care venues a requirement that is buttressed on seamless coordination functions. Studies have highlighted that facilitating inter-organisational and interprofessional care planning for patients is capacitated by mechanisms such as a shared electronic medical record platform and joint clinical case conferences between providers at different care interfaces [30,31]. Harmonisation of cross level service delivery will enable both patients and providers to efficiently navigate within and beyond the province of primary care.

C4: “Continuity of care”, which describes the longitudinal use of a regular care provider, fostering a provider-patient relationship over time [24]. Studies have discerned that continuity of care embraces three main forms, namely relational, informational and managerial which commonly results in longitudinal and personal continuity between GPs and patients [32]. A strong patient-GP relationship is instrumental in improving the uptake of preventive care and enhance adherence to treatment. On the contrary, a lack of connected and coherent care had been shown to be associated with a higher risk of mortality, driving the need to build strengthen therapeutic relationships between patients and their GPs [33]. This is particularly so for patients with chronic conditions like diabetes, where constant monitoring and follow-up with a care provider can potentially circumvent further complications [34].

Since Starfield first articulated them in 1992, the “4Cs” have been used to design and plan primary care systems and develop novel ways of measuring primary care models such as the Patient-Centered Medical Home in the United States and Canada [35,36,37]. After the first “4Cs” were well-established, other similar paradigms started emerging, using the original “4Cs” as cornerstones. The less well-known “10Cs”, which include competence, cost-effectiveness, communication, collaboration, compliance and competing interests, were, to an extent, an offshoot from their parent framework [38].

As primary care took centre stage in most health systems, an expanded version of the “4Cs” framework was developed, pivoted to evaluate the Patient-Centered Medical Homes. This became coined as the “9Cs” of accountable primary care, which moved beyond the medical home model. Again, additional attributes such as physician creditability, collaborative learning, cost-effectiveness, capacity expansion and career satisfaction were added to Starfield’s original pillars of exemplary primary care [39].

Notably, other primary care frameworks were also employed to assess the performance of similar primary care structures. For example, in Bodenheimer’s ten building blocks of high performing primary care, a practical conceptual model was employed to evaluate PCMH. The model’s foundation was built on Starfield’s “4Cs”, with other building blocks such as engaged leadership, data-driven improvements and patient-team partnerships that support the four essential functions of primary care [40]. On the ground evaluation of a primary care system necessitates a practical evaluation tool, in this case, the Primary Care Assessment Tool (PCAT), a publicly owned instrument implemented by the World Health Organization that empirically measures essential primary care attributes, which are organised around the fields embraced by Starfield’s “4Cs” while capturing other salient domains, such as family orientation, community associations and cultural relevance [41]. Fundamentally, Bodenheimer’s ten building blocks and PCAT, both of which are widely accepted and adopted, have the “4Cs” at their nexus, making Starfield’s framework a suitable and validated one to be deployed for our research.

With the advent of an increasing chronic disease burden, pathways to health system gains through cost containment, better health outcomes and more equitable access are widely accepted to be attainable through a primary care system which enshrines Starfield’s “4Cs”. To our knowledge, there are only two published quantitative studies conducted to date that evaluates the effectiveness of PCN in Singapore, both exclusively for diabetes management, and no prior study with an emphasis on mapping the characteristics of the PCN to the “4Cs” framework had been conducted despite the recent promulgation of the PCN [42,43]. To fill the gap in research, this secondary qualitative analysis study aims to map the operational characteristics of PCN to Starfield’s “4Cs” of high performing primary care and, in doing so, provide deep understanding of how PCN empowers GPs to manage patients with chronic conditions through the attainment of the “4Cs”.

## 2. Methods

### 2.1. Study Design

A secondary qualitative analysis was performed using qualitative data obtained from an earlier study exploring the perceived barriers and facilitators experienced by private GPs enrolled in PCNs [44]. Consequently, it was considered valid to re-examine the collected data and explore how PCN manifests the “4Cs” when managing patients with chronic conditions [45]. We used supplementary analysis, a specific type of secondary qualitative analysis for this study, which helps address aspects of data which were either not addressed or only partially addressed in the original study. In turn, enabling the research team to thematically analyse the data set from a different angle [46].

### 2.2. Sample

As the focus of this study was on mapping the operational characteristics of the PCN, we purposefully selected the transcripts of all the GPs (*n* = 30) who were involved in the original study. Within our sample, three were female and 27 were male. Our participants’ age ranged from 31 to 68 years, with an average of 49 years and years of experience practising in primary care ranged from 3 to 35 years, with an average of 18 years. In addition, participants were recruited from 8 out of 10 PCNs in Singapore.

### 2.3. Recruitment and Data Collection of the OriginalSstudy

Participants from the original study were recruited using purposive and snowball sampling techniques. The inclusion criteria of sample necessitated that participants had to be private GPs enrolled in a PCN and practising at the time of interview. Prospective participants were contacted using their contact details such as email addresses and clinic telephone numbers which were made publicly available on public domain websites. For the original study, the interviews which were audio-recorded were conducted at a conducive location after signed informed consent was obtained from the participants. The interview guide used was piloted tested with two GPs before implementation. The feedback obtained from the pilot tests were used to iteratively modify the interview guide in terms of the questions’ objectivity, brevity and tone, such that they could adequately draw relevant and in-depth responses from the main pool of participants when formally implemented. Overall, the interview guide comprised of questions on the present status of Singapore’s primary care landscape and the PCN (Supplementary Material: Topic guide). No reimbursement was provided for participation in the study. Thirty private GPs participated in our interviews lasting 40 to 90 min and detailed field notes were also collected during the interviews. All interviews (which were conducted in English) were transcribed verbatim by a professional transcriptionist and assessed for accuracy by the study team.

After the interviews, the audio recordings and subsequently, audio transcripts were anonymised by removing potentially identifying information such as names of clinics and GPs and replacing them with pseudonyms to ensure that the data could not be traced to the participants. Member checking, whereby interview transcripts were sent back to participants to be reviewed, was not performed. Consolidated criteria for reporting of qualitative research (COREQ) checklist was used throughout this study (Supplementary Material: Table S1) [47].

### 2.4. Ethics Approval

Ethics approval was obtained from the National University of Singapore, Institutional Review Board (NUS-IRB) before starting the study. The NUS-IRB reference code is S-19-005.

### 2.5. Data Analysis

For this study, using Starfield’s “4Cs” conceptual framework, we performed a framework analysis of the transcripts to derive themes salient to the “4Cs” of primary care. This procedure allowed us to map the operational features of the PCN to the criteria set forth by Starfield’s primary care components. To ensure rigor, research team members from the original study (*n* = 4), as well as new research team members (*n* = 2) with fresh perspectives uninfluenced by the findings of the first study, were involved in the secondary qualitative data analysis using uncoded transcripts from the original study.

Transcripts were analysed inductively using thematic analysis, and deductively using Starfield’s ‘4C’ framework with NVivo version 12 for Windows software (QSR International Pty Ltd. Version 12, Ottawa, ON, Canada). We followed a five-step framework analysis approach proposed by Ritchie and Spencer to allow themes that were relevant to the “4Cs” to surface [48]. In brief, we first familiarised ourselves with the transcripts, coded aspects salient to our research question and organised codes into themes, namely the “4Cs”, so as to map the codes and themes to domains featured in Starfield’s framework. In order to increase trustworthiness, the research team would raise and discuss any disagreements regarding the themes and codes until a consensus was reached. To further improve inter-rater reliability, the same five-step approach was employed by all coders. Thereafter, final themes and codes were agreed among all the authors after multiple rounds of iterative feedback and thematic saturation was reached. In addition, the research team drew reference from the field notes collected in the original study to guide the reflexive process and to give context during the coding process.

## 3. Results

### Main Findings

**Figure 1:**
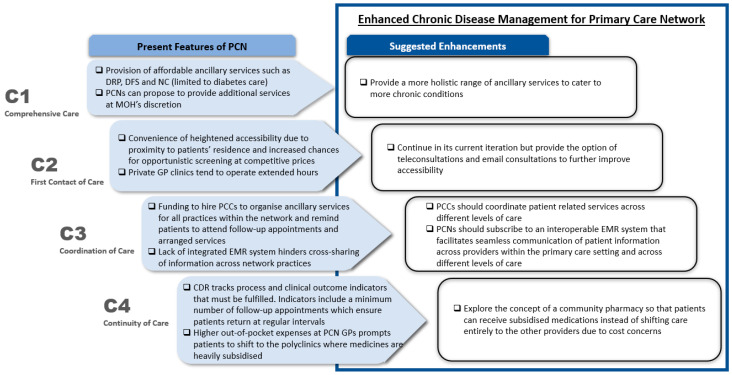
Aspects of PCN that achieved the “4Cs” and suggested enhancements derived from analysis of 30 transcripts from interviews with GPs. DRP—diabetes retinal photography; DFS—diabetes foot screening; NC—nurse counselling; PCN—primary care network; MOH—Ministry of Health; GP—general practitioner; PCC—primary care coordinator; EMR—electronic medical record; CDR—chronic disease registry.

Our results showed that the PCNs did fulfil most of the criteria set forth by Starfield’s “4Cs” and have summarised them in Figure 1 below. The various aspects of the “4Cs” and suggested enhancements will be elaborated in our discussion.

C1: Comprehensive care. For every PCN, ancillary services were provided around the premises of the GP clinics, which were traditionally not made available to them due to the lack of economies of scale to provide services at low cost to patients. In addition, PCNs were free to choose a model of service delivery that suited their operational needs, and our data showed that the most common mode of service provision was through a roving team of nurses. Each practice was equipped with the capacity to offer such services, transforming these practices into a “one-stop-shop” for consultation, health screening and self-management education. The mandated ancillary services for each PCN were limited to diabetes mellitus care such as diabetic retinal photography (DRP), diabetic foot screening (DFS) and nurse counselling (NC). DRP and DFS detected early signs of diabetes progression while NC offered education to patients on disease-modifying behaviours and self-management strategies, thus offering timely interventions for patients at these clinics and in turn preventing the deterioration of patients’ conditions.

“I am able to offer a one-stop service for my diabetic patients. So, when they come here [PCN GP Clinic], they will have their DRP, DFS done and they come for consultation with the doctor and even for nurse counselling. It is all done at that one stop [PCN GP Clinic]. So, the patients actually like it and there is very little reason for them to want to go elsewhere.”

(R48)

The PCNs also operated on an amorphous model which enabled the provision of additional services, such as spirometry and Holter monitor tests, if proposed by the PCN and subsequently authorised based on MOH’s discretion.

“At the moment, I think they [MOH] are exploring different means of enhancing primary care, by having the spirometry, the Holter test which I have not used. […] for patients that need all these, initially, we will send to the hospital because we can’t do it. Now if the PCN provides these capabilities at the primary care level, we can do all these. We don’t have to refer them. Now if it is available and the PCN can do it […] I will contact my PCN to arrange for you [the patient], then that raises our capability.”

(R39)

The costs of these ancillary services are decided by each PCN; however, the prices that were charged were lower than market rate (what is charged at the polyclinics) and some PCNs even went as low as $1 to make these services more attractive to their price-sensitive patients.

“We charge a very nominal pricing [for ancillary services] for PCN […] a PG [Pioneer Generation, elderly] patient will pay $10 flat for any of these three services whether it’s eye, foot or nurse counselling. It is just $10.”

(R36)

“Our nurse counselling is $1. So, my patients cannot say no, right? In fact, for example, eye check [DRP], foot check [DFS], are all priced at maybe half to one third of the polyclinic fees. In fact, that is the truth. My patient looked at the polyclinics and said that polyclinics are more expensive than us.”

(R17)

C2: First contact of care. The provision of ancillary services is linked to another essential tenet of good primary care (i.e., first contact), as patients have increased access to ancillary services at their neighbourhood GP clinics without having to travel further to other care venues. With the availability of ancillary services within clinic premises, GPs could also perform opportunistic screening for patients when they present for their acute consultation visits.

“[…] some of them could be seeing us for common illnesses and we will ask them have you had your eye screened before? Then if they say never, I say why don’t you have it done here? So that is where it gives us a chance to have a conversation starter with them.”

(R48)

Furthermore, most GPs operate long clinic hours which extended into the night on weekdays and on weekends unlike their polyclinic counterparts, creating increased accessibility, especially for the working population.

“Some of them [patients] are agreeable [to seek treatment from private GPs] because they can come after office hours.”

(R30)

The PCNs would have nurses from the mobile ancillary services team available on the clinic premises to present the benefits of receiving ancillary services from a PCN clinic. This would include going through the cost benefits of using the PCN’s ancillary services. Having a lower price point as mentioned above will also posture PCN GP clinics as a first choice when coming to receiving these ancillary services.

“We [GPs and PCN nurses] have a little bit more time to explain to them [patients] how it can be very affordable even at the clinic, if not they [patients] will always think that polyclinics are always cheaper. But it is not true you know. But we do not have time to explain to them. So, we have all these nurse counsellors [PCN nurses] where they have a bit more time to interact with the patients [explain the benefits of receiving services from PCN] when they do the diabetic retinal photography and when they do the nurse counselling then they [patients] begin to understand.”

(R48)

C3: Coordination of care. The PCNs received funding from MOH to hire primary care coordinators (PCCs) to coordinate with GPs across the network to arrange for the ancillary services. PCCs also liaise with individual GP practices to coordinate with their patients and remind patients to attend ancillary services and return for follow-up consultations. Thus, the additional manpower provided to the PCNs could facilitate the reduction in patients’ non-attendance for appointments.

“The PCCs will follow up with the patients on their appointments, they will book their appointments and then bring the provider [ancillary service provider] to provide their service in our clinic.”

(R46)

The GP clinics use varied types of electronic medical record (EMR) systems. Hence, the lack of an interoperable EMR system with other GPs within their network impeded patient information sharing across the system, which might lead to fragmented care and miscommunication among providers. Furthermore, some GPs were still using hardcopy records to keep track of their patient information which added to the difficulty in seamless record keeping for the entire network.

“No, there is no common one [EMR], because we are different, different clinics coming from different backgrounds and so on […] so we have to try and get some commonality [same EMR] so that we can draw the information […], so there is a standard.”

(R19)

“About five of them, initially out of the 30 [GP practices] were on paper and pen, but they are now in flight to convert to some form of electronic medical system.”

(R36)

C4: Continuity of care. Every PCN must maintain a chronic disease registry (CDR) that promotes care continuity by tracking process and clinical outcome indicators, such as the number of follow-up consultations, types of ancillary services attended and clinical parameters such as glycated haemoglobin, blood pressure and cholesterol levels. The CDR served as a reminder mechanism for GPs to ensure that necessary tests, ancillary services and follow-ups are performed which would otherwise have been difficult to monitor amid a busy clinical day as a solo GP. This judicious monitoring of patients in turn ensures that the clinical parameters remain within the optimal range for their patients thus preventing potential deterioration and subsequent need for specialist care. The CDR further promoted continuity of care as GPs would receive Care Plus Fee (CPF) (monetary reimbursement of SGD 100 per patient per year) when specific CDR indicators, such as a minimum number of follow-up appointments, were fulfilled within a stipulated timeframe (usually within one calendar year). This quantum was geared to offset the extended consultation time required to manage a chronic patient. This is because the revenue generated by private GP practices is volume-based, making it more profitable for GPs to see more acute cases. However, complex chronic patients require a lengthened consultation. Hence, the CPF was introduced as a way to reimburse clinics for that trade-off in revenue generation.

“[…] CDR reminds especially the private doctors when your clinic so busy, a lot of times we will overlook, or we will you know forget certain things. So, this, in a way, is a constant reminder to make sure that this has been done for the patient.”

(R26)

“Everyone, every clinic, every single clinic in the PCN should be eligible for the Care Plus Fee, if there are patients who satisfy the criteria required. I think three visits per year or two or three visits per year with the necessary blood investigations [amongst other requirements] being done for them to be eligible for the Care Plus Fee.”

(R18)

“It [Care Plus Fee] is basically a process indicator and regularity of follow-up […] that was a carrot [financial incentive] to tell the GPs don’t be afraid to see complex patients […] to recognise the increased time that you [GPs] are spending with complex patients.”

(R36)

In terms of healthcare financing, in the private primary care sector, the Community Health Assist Scheme (CHAS) was implemented as a means-tested finite portable medical subsidy to enable patients to receive medical subsidies at private GPs, thus reducing out-of-pocket payments. However, patients who suffer from multiple chronic conditions require more medications, placing a financial burden on them despite having CHAS subsidies. These patients tend to return to the polyclinics due to its heavily subsidised medications as compared to the full unsubsidised prices they pay at private GPs. Therefore, the adverse financial gradient between private GPs and polyclinics due to the discrepancy in medication pricing severed patients’ continuity of care with the GPs.

“The CHAS subsidies help, but it is for simple chronic illness. For simple cases, it may be comparable to the polyclinic. But when it comes to more medications, it makes it very difficult, even with the CHAS subsidy.”

(GP R48)

“If you are talking about chronic patients here, then the distribution between private and public chronic patients are highly steered towards the government side [polyclinics] because polyclinics are offering such a high subsidy that it makes no sense for patients to follow up with private [GPs]. Most of the patients that follow up with private [GPs] are simple chronic patients, meaning that they are probably on one or two chronic medications […] So that means out-of-pocket [costs] will not be so high per year. But what happens when the disease starts to deteriorate or what happens when people start to age, and they need more and more medications to control their chronic diseases? Their out-of-pocket [costs] in primary care in private sector is going to get higher and higher until a point where all these patients are driven back to the polyclinics.”

(R26)

## 4. Discussion

Our results showed that the features of the PCN had potential to achieve Starfield’s “4Cs” of primary care, for the management of patients with chronic conditions to an extent. The provision of ancillary services, chronic disease registry and financial incentives capacitate the PCNs to enshrine similitudes of comprehensiveness, first contact, coordination and continuity of care. Unfortunately, the lack of an integrated EMR, insufficient CHAS subsidy quantum and adverse financial gradient favouring polyclinics (all of which fall out of the province of the PCN policy) had made it challenging for PCNs to fully manifest the “4Cs” of primary care. Taking a broad view, our findings also pointed to the delicate interactions of the “4Cs” within and beyond each other in terms of optimising GPs’ delivery of high-value chronic disease care within the sphere of primary care. Hereafter we will elaborate in detail on each “C”, while concurrently arguing the dynamic interplay between them to paint a fuller picture of how the “4Cs” manifest themselves in PCN.

The “4Cs” may either enhance or inhibit one another. For example, providing comprehensive ancillary services at a considerably lower prices at PCN GP clinics, as compared to polyclinics, had enticed patients to view PCN GP clinics as a first option to receive these services, and return for not only their consultations but regular ancillary check-ups as well. These findings corroborate with a study by Ann et al., which highlighted that comprehensive care is associated with improved continuity of care [49]. However, in Singapore, only, DRP, DFS and NC were the mandated ancillary services offered by the PCNs, due to its heightened need as the prevalence of diabetes and its related complications in Singapore were projected to double from 7.3% in 1990 to 15% in 2050 and from 12.2% in 2007 to 24.3% in 2035, respectively [50,51]. Additionally, the total economic burden of managing diabetes is estimated to climb from one billion USD in 2010 to 2.5 billion USD in 2050 [52]. Hence, the Singapore government launched a “war on diabetes” to re-orientate the healthcare system to have a stronger focus on diabetes management [53]. However, the present iteration of the PCN has limited the range of ancillary services to diabetes care, posing a shortcoming to attaining full comprehensiveness, as comprehensive care encapsulates services addressing both breadth and depth of coverage by the primary care team beyond diabetes care [54]. Although diabetes poses a threat to health security and has received rapid prioritisation and funding, other non-communicable diseases should also be covered. Hence, a wider array of services such as physiotherapy and social prescribing is merited for the next iteration of PCN to provide a heightened level of comprehensiveness [55]. Another example is, before enrolling into PCNs, GPs used to coordinate care with ancillary service providers by arranging appointments, referrals, and following up of test results. However, being able to offer comprehensive ancillary services within the clinic premises enable less care coordination events by GPs and concurrently enhances patients’ convenience. Additionally, providing a comprehensive range of services is found to nurture relationship continuity. These findings are supported by a study conducted by Freeman et al., which showed that providing a range of ancillary services, phone consultations, advance appointments and quality of consultation were crucial to securing relationship continuity [39,56]. However, it is also worth noting that a highly comprehensive clinic may involve multiple providers, which in turn could diminish the patient’s sense of continuity. Care coordination for PCNs in Singapore is facilitated by PCCs with a more limited role than individuals (usually called care navigators) in other contexts [57]. Rather than managing patients’ follow-up services across different levels of care (i.e., between specialist units and primary care clinics), within the purview of the PCN, PCCs manage patients exclusively within the ambit of the primary care interface. However, small steps pave way to big changes. The provision of PCCs has fostered integration of care within the private GP sector, which had otherwise been fragmented, where GPs tend to practice in siloes. Additionally, an interoperable EMR system, teleconsultations and email consultation should be made available at all facets of care to nurture care coordination such that patient information can be shared seamlessly across different provider settings. Having an electronic presence can make PCN GPs more palatable to patients to use them as their first point of care and continual care for non-emergent and follow-up consultations [58,59,60].

First contact increases patients’ possibility to see the same healthcare provider and in turn enhance care continuity. Studies have shown that patients rank care continuity as the highest among the other attributes of primary care because a long-standing relationship helps to build trust [61,62]. Having built a trusting relationship through care continuity with their GPs, patients will be more willing to adhere to physician recommendations [38]. One way to enforce care continuity is via a gatekeeping mechanism. This concept primarily holds true for some western countries. However, it is not the case for Singapore. The country’s small land area, availability of numerous healthcare facilities island-wide, a highly efficient inter-connected transportation system and historical preference of patients seeking primary care services with a provider of their choice could be reasons for not having a mandated gatekeeping role for GPs [63,64]. On the other hand, studies have shown that patients develop relational discontinuity with their GPs when care continuity is enforced via gatekeeping. This is because a mandated gatekeeping mechanism potentially limits patients’ healthcare choice to a single primary care provider and might result in delayed diagnosis and treatment [56]. Our study findings illustrated that patients developed relational discontinuity with their GPs due to the adverse financial gradient between the public and private primary care sector. Inevitably, first contact and care continuity at the private primary care interface are disrupted because of this. Patients with complex chronic conditions requiring multiple medications were consequently prompted to return to the heavily subsidised polyclinics to enjoy lower out-of-pocket expenses. More importantly, without cultivating a long-standing trusting relationship with the GPs, patients will want to seek care from tertiary hospitals or polyclinics even for health problems within the realms of the GP sector [39]. While it is crucial to strengthen continuity of care, instead of enforcing a gatekeeping mechanism, other approaches can be explored. For example, the adverse financial gradient between the public and private primary care sector can be partially surmounted by developing community pharmacies whereby patients obtain government-subsidised medications after consulting a PCN GP. Moreover, educating patients on the value and advantages of seeking the same primary healthcare provider can be promoted by the PCNs and also made more cognizant at the policy level.

## 5. Conclusions

The implementation of the PCN is highly pragmatic and timely against the looming backdrop of an ageing population. Therefore, our study findings provide evidence on the capacity of the PCN to confer enhanced chronic disease management capacity to GPs by sufficiently meeting the criteria of Starfield’s “4Cs” through the provision of a suite of ancillary services, heightened financial and physical accessibility to services at GP clinics, manpower to coordinate between practices and liaise with patients for follow-up and a registry to ensure care processes are diligently fulfilled. Even though certain aspects such as the lack of a common EMR system, insufficient government subsidies at private GPs and adverse financial gradient favouring public primary care require refinement, the empowering features mentioned above outweigh the shortfalls in all important aspects of providing optimal chronic disease management in a primary care setting.

While this analysis was limited to data collected from the previous qualitative study [44], the data were suitably rich to allow us to gain meaningful new insights. Moreover, this study’s research question fitted well with that of the original study, as both studies were concerned with chronic disease management in PCNs. However, as data were limited to the providers’ perspective, we propose a future study to understand how features of the PCN fit well with Starfield’s “4Cs” framework from the patients’ angle. Integrating patients’ perspective to tweak health delivery models unlocks opportunities to improve patient satisfaction, patient experience and overall health outcomes [65]. Therefore, though still in its early stage of development, PCN has proven to be well positioned to drive private GPs towards enhanced chronic disease management.

## Data Availability

The data presented in this study are available on reasonable request from the corresponding author.

## Supplementary Materials

The following are available online at https://www.mdpi.com/1660-4601/18/6/2926/s1, Supplementary material: Table S1; Supplementary material: Topic guide.
