# Supplementary material for: Primary Care Networks and Starfield’s 4Cs: A Case for Enhanced Chronic Disease Management
Source: Int J Environ Res Public Health. 2021 Mar 12;18(6):2926. doi: 10.3390/ijerph18062926 (PMC8001119; doi:10.3390/ijerph18062926)
Supplement: Supplementary file 1 [file ijerph-18-02926-s001.zip › Supplementary material topic guide.docx]

**Supplementary material: Topic guide**

| **Section I**: Introductory questions |
| --- |
| 1. To start, can you tell me more about yourself?  - Can you share more about your professional background? Current role/roles and job scope? - Can you describe the organisation/s and capacities of the PCN? (prompt: composition, structure and history of the organisation/entity) - Can you share about your educational background (i.e. Graduate Diploma in Family Medicine, Master of Medicine in Family Medicine, the Collegiate Membership of the College, Fellowship of the College, etc.)? |
| **Section II**: Current Status of Primary Care in Singapore |
| 1. What is your definition of primary care? 2. How does the functioning of the PCN facilitate primary care in Singapore? 3. How did the current primary care schemes such as PCN come about? 4. What do you think are the major advantages of the current primary care system with regards to the PCN? Why? (prompts: funding, ancillary services, administrative support, team-based care etc.) 5. What do you think are the major disadvantages of the current primary care system with regards to the PCN? Why? |
| **Section III**: Future of Primary Care in Singapore |
| 1. How do you think primary care will evolve over the next few years? *(prompt: What do you think will change? What forces will drive the change?)* 2. What do you hope primary care will look like over the next 5 to 10 years? |
| Closing questions and remarks |
| 1. Before we end, do you have any final thoughts to share? 2. We have come to the end of the interview. Do you have any other questions? |
