# Supplementary material for: Primary Care Networks and Starfield’s 4Cs: A Case for Enhanced Chronic Disease Management
Source: Int J Environ Res Public Health. 2021 Mar 12;18(6):2926. doi: 10.3390/ijerph18062926 (PMC8001119; doi:10.3390/ijerph18062926)
Supplement: Supplementary file 1 [file ijerph-18-02926-s001.zip › Supplementary material table S1.docx]

**Supplementary material: Table S1**

| **DOMAIN 1: RESEARCH TEAM AND REFLEXIVITY** | |
| --- | --- |
| ***Personal characteristics*** | |
| 1. Interviewer/facilitator | CD and SS conducted the interviewes. |
| 2. Credentials | CD and SS both hold a MPH.  GJ holds a MA.  JA holds a PhD.  DM holds an MD.  GK holds a MBBS and a PhD. |
| 3. Occupation | CD, SS and GJ are research associates in Singapore.  JA, DM and GK are professors in Singapore.  DM is also a professor in the United States. |
| 4. Gender | CD,GJ, JA, DM and GK are male and SS is female. |
| 5. Experience and training | All team members had prior experience and training in qualitative research methods. |
| ***Relationship with participants*** | |
| 6. Relationship established | All team members had no prior relationship with the participants. |
| 7. Participant knowledge of the interviewer | All team members had no prior relationship with the participants. |
| 8. Interviewer characteristics | All team members have extensive experience in the field of primary care and family medicine.  All team members have also resided in Singapore for more than 5 years, giving them sufficient knowledge of the local context and nuances in the health system. |
| **DOMAIN 2: STUDY DESIGN** | |
| ***Theoretical framework*** | |
| 9. Methodological orientation and theory | Secondary qualitative analysis approach using data obtained from semi-structured in-depth interviews with GPs enrolled in PCNs.  Starfield’s ‘4Cs’ framework was employed to map the operational features of the PCN to each ‘C’ explicated in the framework. |
| ***Participant selection*** | |
| 10. Sampling | Purposive and snowball sampling techniques were used to recruit GPs who were practising in a PCN during the time of interview. |
| 11. Method of approach | Purposive and snowball sampling techniques were employed to recruit GPs who were practising in a PCN during the time of interview.  For purposive sampling, we used the contact details of eligible GPs available from public domain websites to reach out to them.  For snowball sampling, GPs whom we had interviewed would refer us to their colleagues who also met our inclusion criteria. |
| 12. Sample size | A total of 30 participants were recruited. |
| 13. Non-participation | 7 GPs had refused participation citing insufficient time to be interviewed.  In addition, no reimbursement was provided for participating in our study. |
| ***Setting*** | |
| 14. Setting of data collection | A place that was deemed conducive to conduct the interview with minimal distractions and of convenience to the participants. |
| 15. Presence of non-participants | Not present. |
| 16. Description of sample | Participants were practising GPs enrolled in a PCN who had consented to take part in this study.  A total of 30 GPs were recruited, out of which 27 were male, and three were female with an average practising duration of 18 years in primary care.  Most of the participants are ethnically Chinese. |
| ***Data collection*** | |
| 17. Interview guide | The interview guide was piloted tested with 4 GPs and changes made to the guide before actual implementation with our 30 participants.  The interview guide can be found as supplementary material 1. |
| 18. Repeat interviews | Repeat interviews were not conducted. |
| 19. Audio/visual recording | We audio-recorded the interview after seeking the participants’ consent to be recorded. |
| 20. Field notes | The field notes were collected by SS while CD conducted the interviews.  This procedure was repeated for all the interviews.  The field notes were used during the data analysis process to provide contextual information and simultaneously guide the reflexive process. |
| 21. Duration | Interview duration ranged from 40 to 90 minutes. |
| 22. Data saturation | Data saturation was achieved after no new themes and subthemes emerged from our data. |
| 23. Transcripts returned | Member checking not performed, but the participants were given the option to review their transcripts but was not taken up by any participant. |
| **DOMAIN 3: ANALYSIS AND FINDINGS** | |
| ***Data analysis*** | |
| 24. Number of data coders | All team members were involved in the data analysis process, and a standard protocol for analysis was closely adhered to promote inter-rater reliability. |
| 25. Description of the coding tree | The main findings are summarised in figure 1.  The figure depicts the main themes that emerged, with reference made to the ‘4Cs’. |
| 26. Derivation of themes | The themes and subthemes were derived from the data using a secondary qualitative analysis approach while using Starfield’s ‘4Cs’ framework to elucidate themes that were salient to the ‘4Cs’.  We also employed an iterative process to discuss the derived codes and themes to ensure that the finalised results agreed among all team members were accurately reflective of the data set and research question. |
| 27. Software | QSR NVIVO version 12 was used throughout the analysis process. |
| 28. Participant checking | Member checking not performed, although the option of reviewing the transcripts was given to the participants. |
| ***Reporting*** | |
| 29. Quotations presented | Yes, they are presented in our results section. |
| 30. Data and ﬁndings consistent | Yes, they are presented in our results and discussion sections. |
| 31. Clarity of major themes | Yes, they are presented in our results and discussion sections. |
| 32. Clarity of minor themes | Yes, they are presented in our results and discussion sections. |
